# Improving the efficacy of enuresis alarm treatment through early prediction of treatment outcome: a machine learning approach

**DOI:** 10.3389/fruro.2023.1296349

**Published:** 2023-11-24

**Authors:** Karl-Axel Jönsson, Edvin Andersson, Tryggve Nevéus, Torbjörn Gärdenfors, Christian Balkenius

**Affiliations:** ^1^ Department of Biomedical Engineering, Lund University, Lund, Sweden; ^2^ Department of Women’s and Children’s Health Uppsala University, Uppsala, Sweden; ^3^ Pjama AB, Skurup, Sweden; ^4^ Department of Philosophy, Lund University Cognitive Science, Lund, Sweden

**Keywords:** enuresis, enuresis alarm, random forest, predictions, machine learning, application, data

## Abstract

**Introduction:**

Bedwetting, also known as enuresis, is the second most common chronic health problem among children and it affects their everyday life negatively. A first-line treatment option is the enuresis alarm. This method entails the child being awoken by a detector and alarm unit upon urination at night, thereby changing their arousal mechanisms and potentially curing them after 6–8 weeks of consistent therapy. The enuresis alarm treatment has a reported success rate above 50% but requires significant effort from the families involved. Additionally, there is a challenge in identifying early indicators of successful treatment.

**Methods:**

The alarm treatment has been further developed by the company Pjama AB, which, in addition to the alarm, offers a mobile application where users provides data about the patient and information regarding each night throughout the treatment. The wet and dry nights are recorded, in addition to the actual timing of the bedwetting incidents. We used the machine learning model random forest to see if predictions of treatment outcome could be made in early stages of treatment and shorten the evaluation time based on data from 611 patients. This was carried out by using and analyzing data from patients who had used the Pjama application. The patients were split into training and testing groups to evaluate to what extent the algorithm could make predictions every day about whether a patient’s treatment would be successful, partially successful, or unsuccessful.

**Results:**

The results show that a large number of patient outcomes can already be predicted accurately in the early stages of treatment.

**Discussion:**

Accurate predictions enable the correct measures to be taken earlier in the treatment, including increasing motivation, adding pharmacotherapy, or terminating treatment. This has the potential to shorten the treatment in general, and to detect patients who will not respond to the treatment early on, which in turn can improve the lives of children suffering from enuresis. The results show great potential in making the treatment of enuresis more efficient.

## 1 Introduction

Bedwetting, or enuresis, is a very common chronic health issue among children. Studies indicate that approximately 5% to 10% of 7-year-olds experience enuresis, and for a subset of individuals this problem can persist into their teenage and adult years (1). Enuresis substantially impacts children’s wellbeing, leading to challenges such as diminished self-esteem, increased anxiety, and heightened stress levels (2).

There are currently two main available methods for treating enuresis: alarm treatment and using the antidiuretic medication desmopressin. The enuresis alarm is a well-established initial treatment for enuresis (1). The principal objective of this therapeutic approach is to gradually modify the sleep and arousal of the child by consistently awakening them immediately when their bladder is emptied during sleep. This is achieved through the utilization of a sensitive urine detector that promptly triggers a distinct audible signal upon the appearance of the initial urine droplet. The efficacy of the alarm treatment exhibits considerable variation across various studies, typically ranging from 50% to 80% (3–7) and most children that are successfully treated can be considered cured (1).

As per the guidelines outlined by the International Children’s Continence Society (ICCS), treatment with the enuresis alarm should be discontinued if no positive effects are observed within 6–8 weeks. In the event of progress during this time frame, the treatment should be continued until the child achieves a streak of 14 consecutive dry nights (1). If, after a duration of 16 weeks, the child fails to reach this milestone, the treatment can be deemed partially successful if there is a reduction of 50% or more in the frequency of wet nights per week compared with the baseline measurements. Otherwise, it is classified as an unsuccessful treatment (8).

To achieve a successful treatment, adherence to certain key factors is crucial. First, consistent nightly use of the alarm is highly important. Second, having a parent sleep near the child and promptly respond to the alarm is necessary, as children often do not wake up on their own, especially during the initial weeks of treatment. Last, regular support from an instructor is essential to provide encouragement and address any technical issues that may arise. Both the motivation of the child and the family play a crucial role in the treatment’s effectiveness (1).

There are several disadvantages with the alarm treatment of enuresis. First, treatment for 6–8 weeks will, for numerous families, be challenging due to the alarm waking the whole household and the need for one parent to sleep in the same room as the child (1). Consequently, there are issues with treatment adherence (7). Another downside is that there is a relative lack of predictors for successful treatment (9). This is especially problematic for patients who have to undergo 6–8 weeks of treatment without any positive results. Ideally, these patients should be detected early and be given an alternative or parallel treatment. Due to these circumstances, there is substantial interest in identifying the predictive factors for treatment outcomes and shortening the period needed before evaluation regarding continuation of the treatment is carried out.

The use of artificial intelligence in medicine is already widespread in numerous medical fields (10). The objective of this study was to explore the potential of utilizing artificial intelligence with enuresis data collected from a mobile application developed by Pjama Inc, a company specializing in enuresis alarm treatments. The aim was to assess whether the use of AI could help shorten the evaluation period of enuresis alarm treatment. Similar research has been conducted previously in Tokar et al.’s “Application of Machine Learning Techniques for Enuresis Prediction in Children” (11), and in Franco et al.’s “Initial outcomes using a novel bedwetting alarm (Gogoband^®^) that utilizes real time artificial intelligence to wake users prior to wetting” (12). What distinguishes our research from these studies is that it covers the usage of AI during the treatment process, enabling early predictions of the enuresis treatment outcome. This requires a dynamic approach to data collection and machine learning (ML) models, due to the continuous refinement of the model throughout each day of the treatment process.

Random forest is a popular ensemble learning algorithm in ML. Ensemble learning refers to algorithms making predictions from more than one model, and the random forest does this through the combination of multiple decision trees (13). Decision trees are also an established method used for classification in ML, but in larger datasets, where overfitting may be an issue, random forest is preferred due to the variation offered by it (14, 15). Each tree is constructed using a random subset of features and samples of the patient data frame to create diverse sets of trees that are less likely to be correlated and more likely to capture different aspects of the underlying pattern with minimum redundancy. The final predictions are obtained through the algorithm aggregating the predictions of all the individual decision trees (14).

In machine learning, a hyperparameter is a parameter assigned a value before the algorithm runs, thereby influencing the results (16). In random forest some important hyperparameters include: the numbers of decision trees, how deep each tree can grow, and how many features are used for each split. For example, if the numbers of trees and features and the depths are large, the model is more likely to be overfitted to the training data, and if they are too small, the model will be underfitted (17). Therefore, it is crucial to validate the performance and hyperparameters of the model when optimizing it.

K-fold cross-validation is a popular technique for evaluating ML models (18). The algorithm divides the training set into “k” subsets, with one subset serving as test/validation data and the others serving as training data. The process repeats “k” times, training and validating the model in different subsets (17). Analyzing metrics such as mean accuracy and standard deviation across these iterations provides insights into the model’s real-world performance and enables measurement of the randomness of ML pipelines. This helps detect the issues arising from imbalanced or mishandled datasets, which may lead to overfitting or underfitting. To enhance this approach, K-fold cross-validation can be combined with grid search (17). Grid search allows the testing of various hyperparameter values set by the user. For instance, when “k” is 5, the training data split into five parts, and grid search was used to test the hyperparameter combinations across these five data segments. Evaluating the mean accuracy and observing hyperparameter behavior across splits and combinations offers a comprehensive overview of model performance. This also highlights the consistent hyperparameter values with favorable outcomes (19).

## 2 Materials and methods

This study is based on enuresis data provided by the Pjama mobile application, which consists of the data of 3,649 patients who had undergone enuresis alarm treatment. The patients involved in the study were both those who received the enuresis alarm from pediatric outpatient wards in Sweden and individuals who self-initiated the alarm treatment without healthcare supervision. The patients utilized a body-worn alarm in conjunction with the Pjama mobile application to record essential data during their treatment. Upon registering in the Pjama application, patients were required to answer 16 mandatory questions and input specific information before commencing the treatment process. These questions were chosen thoughtfully; they have either been proven to be of significance in the decision of treatment outcomes or are highly suspected to be significant. Some questions were also chosen since they are warning signals for other medical problems, and could lead to the patient being advised to seek medical care instead. The questions were age, sex, type of alarm, how heavy the child’s sleep was, the usual frequency of enuresis, if the child had incontinence issues during the daytime, if they had problems with urgency, if the child had previously tried any treatment (medication and/or alarm), if the child took relaxing medication for the bladder, if they had contact with a nurse, how high their motivation was, if they usually experienced enuresis once or twice per night, if the child was very thirsty, if the child had a weak beam when urinating, and finally, if the child had previously been dry at night.

In addition to the data from the registration, data were continuously gathered during the treatment process. Each day data regarding whether the night was dry or wet were collected, in addition to responses to other questions that provided significant data about the previous night. These questions were related to the severity of wetness, the method of awakening (whether through the alarm or parental intervention), and if any subsequent instances of bedwetting occurred during the same night. If the night had been dry, the question of if the child had woken up to urinate was asked. The time of the enuretic events was also registered by the application.

Out of the 16 questions asked during registration, 26 registration variables were generated. Due to the addition of questions throughout the data collection process from patients, some patients had missing values for specific data points. To address this issue, two approaches were employed. Either the median or the average for a given variable was used, and these were calculated using the responses from other patients who had provided data. Alternatively, a neutral value was used, as for the question about how difficult it was to wake the child, where the answer “don't know” was used.

Based on the information recorded from users each night, 12 different variables describing the events of that night were assigned values. Examples of these variables include whether the night was wet or dry, whether the child wet the bed more than once, or whether data were missing for that particular day.

From the entirety of the data that users input on a daily basis, 14 computational variables were created for each day and each patient. The primary objective of these variables was to enable the model to capture relationships across different days during the course of treatment. Illustrative examples of these computational variables were the count of wet nights a patient experienced in the last 14 days, the number of missed days in the last 14 days, and the number of wet nights experienced in the current week divided by the number of wet nights experienced in the first week. As with the registration data, missing and unreasonable data were treated. For most computational variables, compensatory measures were implemented in instances of missing data, ensuring that the absence of data for a specific day was not simply disregarded. For instance, this was carried out when calculating the number of wet nights during the last 14 days, as there were days within this time frame for which no data were available. In this case the missing nights was seen as neither dry or wet, and an expected value based on the preceding days of treatment was calculated instead. Measures were also taken to filter out unreasonable data, such as when the parent reported that the child wet the bed 17 hours after going to sleep, and replacing these with a mean or median.

After having undergone treatment, the patients were marked as successful, partially successful, unsuccessful, or as dropouts based on the ICCS guidelines. The patients were marked as to what treatment result they had after 8 weeks of treatment. According to the current guidelines for enuresis treatment, a patient should have 14 consecutive dry nights for the treatment to be considered successful (1). To avoid overlooking patients who may have attained dryness but occasionally failed to record their daily status, 2 missing days were deemed tolerable, provided that the remaining 12 days were dry. A dropout was defined as a patient who had not fulfilled at least 6 weeks of treatment, and who had not managed to be dry for 14 consecutive nights before this time. The exclusion criteria for patient selection were if a patient had had 14 consecutive missing days prior to week 8, and that they were not successful in this period. They were then marked as a dropout and excluded from further analysis. A patient that was not marked successful or as a dropout was classed as either partially successful or unsuccessful. To decide which of these applied, the first 2 weeks of the patient’s treatment were compared with the last 2 weeks. If the number of the wet nights experienced had decreased by 50% or more when looking at the last 2 weeks, the patient was considered to be partially successful. The patient was otherwise marked as unsuccessful. The patients used for analysis included all those who were not marked as dropouts.

**Figure 1:**
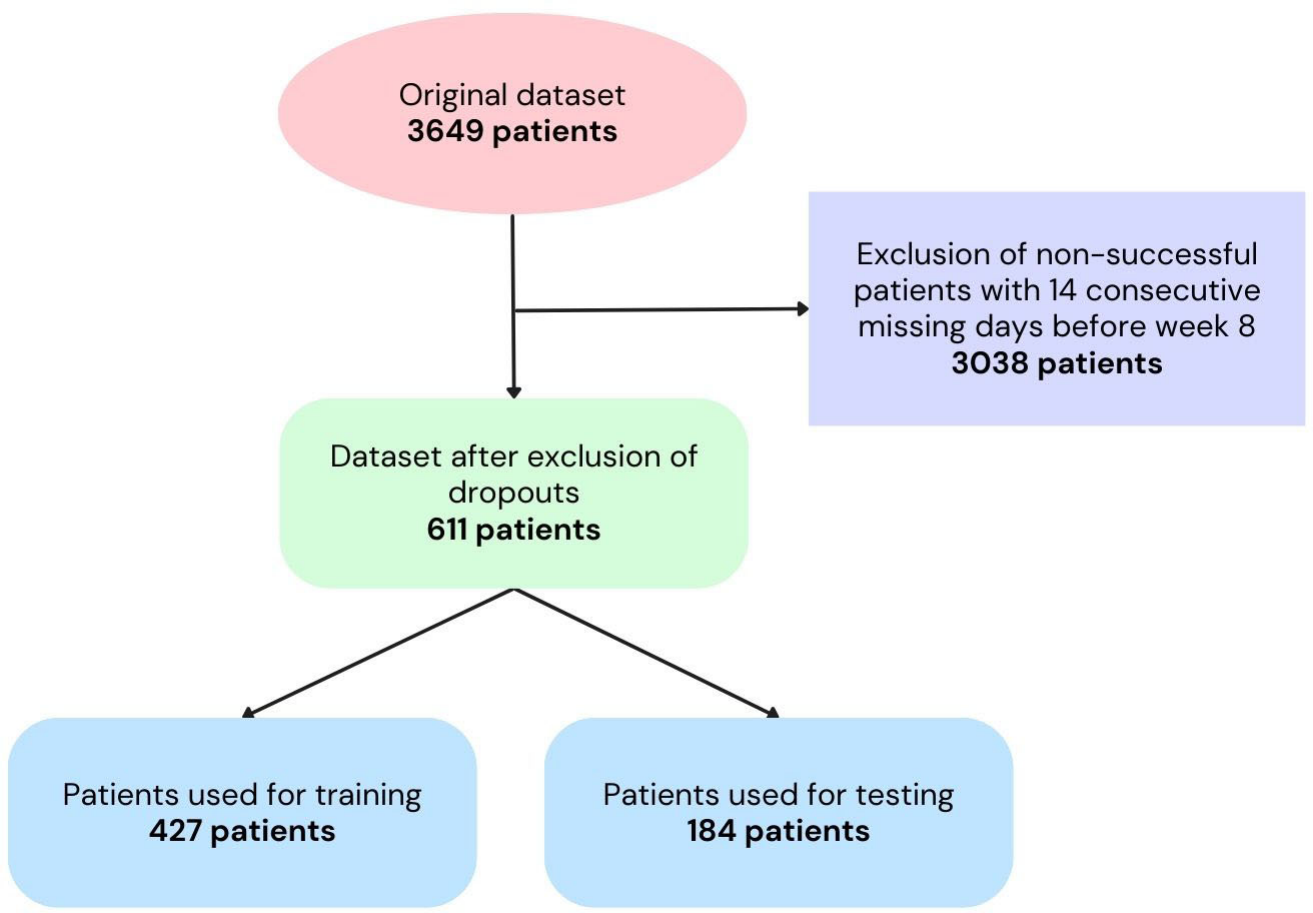
Flowchart of the patient selection process.

The data from 3,649 patients who utilized the Pjama application were analyzed. Out of this initial pool, 611 patients were identified as non-dropouts and subsequently included in the study. Within this subset, 70% of patients were then assigned to the training group, while the remaining 30% of patients constituted the testing group. A visual representation of the patient selection process can be found in **Figure 1**.

The distribution of the variables generated when patients initially registered in the Pjama application for the training and test groups and for all patients is presented in **Table 1**.

**Table 1:** Distribution of registration variables.

	Train	Test		Total
	(*n* = 427)	(*n* = 184)		(*n* = 611)
**Age**	8.47 ± 4.19	8.72 ± 4.61		8.55 ± 4.32
**Sex** (male/female)	73.77% / 26.23%	82.42% / 17.58%		76.61% / 23.39%
**Wet once or twice** (once/twice)	95.09% / 4.91%	97.29% / 2.71%		97.75% / 2.25%
**Motivation** (1–5)	4.05 ± 0.36	4.05 ± 0.51		4.05 ± 0.47
**Alarm type** (alarm with pants/Pjama connect/alarm with underwear/other alarm/no or unknown alarm)	53.39% / 17.56% / 5.38% / 22.24% / 1.43%	49.46% / 15.76% / 5.98% / 28.26% / 0.54%		52.21% / 17.02% / 5.57% / 24.06% / 1.14%
**How heavy the child sleeps** (almost impossible to wake up/difficult to wake up/neither easy or difficult to wake up/easy to wake up/don’t know)	10.77% / 52.22% / 22.48% / 3.75% / 10.78%	8.15% / 26.1% / 9.23% / 50% / / 6.52% /		9.98% / 51.55% / 23.57% / 4.58% / 10.32%
**Enuresis frequency per week**	5.05 ± 1.93	5.18 ± 1.88		5.09 ± 1.91
**Bladder-relaxing medicine** (yes/no)	3.28% / 96.72%	4.89% / 95.11%		3.76% / 96.24%
**Alarm treatment before** (yes/no)	25.60% / 74.40%	25.00% / 75.00%		25.43% / 74.57%
**Medication treatment before** (yes/no)	40.34% / 59.66%	41.28% / 58.72%		40.61% / 59.39%
**Sudden urination in the daytime** (yes/no)	47.58% / 52.42%	48.26% / 51.74%		47.78% / 52.22%
**Wet during day** (yes/no)	13.04% / 86.96%	17.44% / 82.56%		14.33% / 85.67%
**Contact with nurse** (yes/no)	52.05% / 47.95%	63.16% / 36.84%		54.35% / 45.65%
**Weak beam** (yes/no)	3.07% / 96.93%	2.73% / 97.27%		2.97% / 97.03%
**Thirsty at night** (yes/no)	10.17% / 89.83%	10.38% / 89.62%		10.23% / 89.77%
**Dry before but now wet** (yes/no)	16.31% / 83.69%	13.66% / 86.34%		15.51% / 84.49%

**Figure 2:**
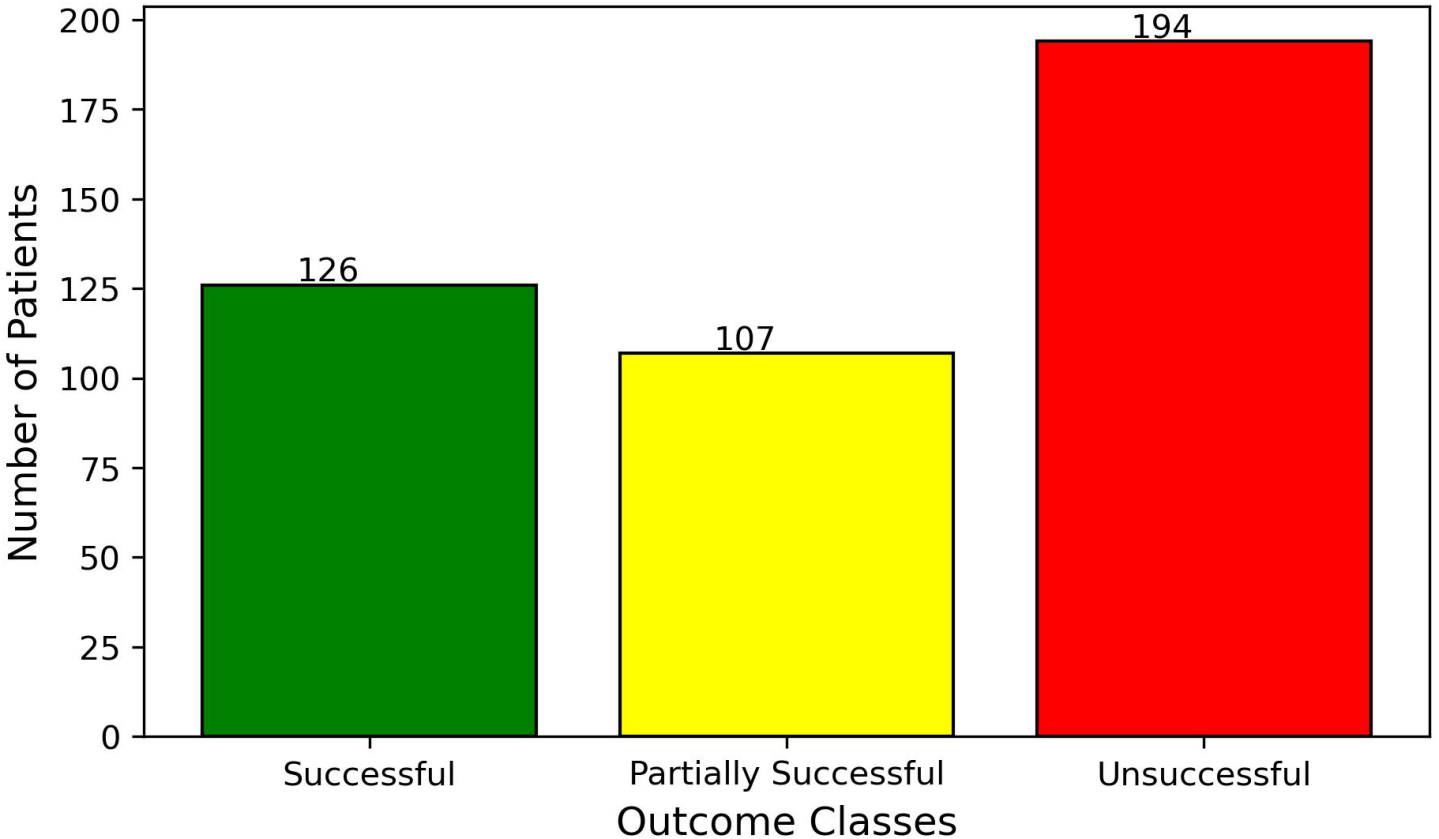
Distribution of patient treatment outcomes within the training set.

**Figure 3:**
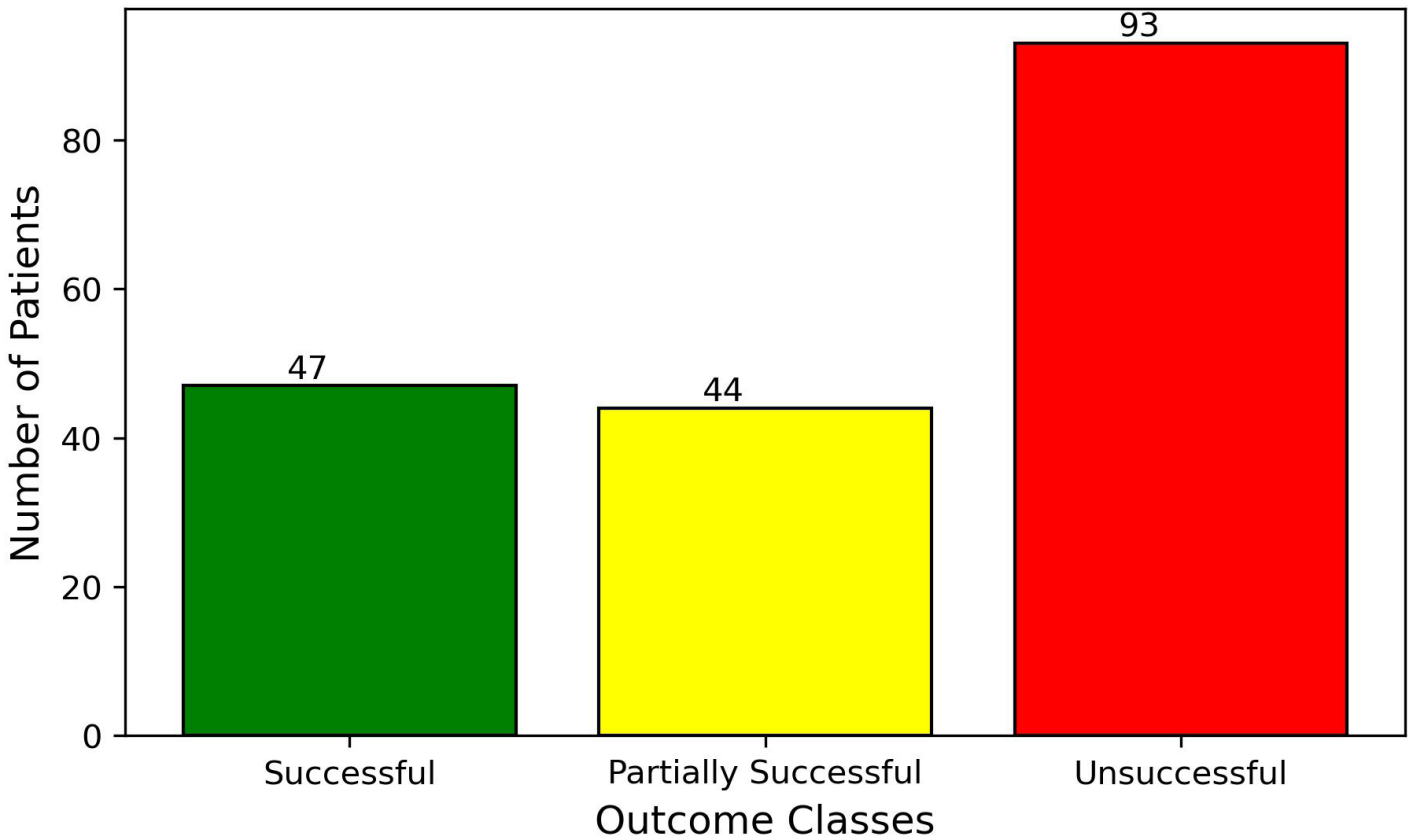
Distribution of patient treatment outcomes within the test set.

The training data consisted of 126 patients who experienced successful treatment, 107 patients with partially successful outcomes, and 194 patients for whom the treatment was unsuccessful. The test data consisted of 47 patients who experienced successful treatment, 44 patients with partially successful outcomes, and 93 patients for whom the treatment was unsuccessful. The distributions are presented in **Figures 2**, **3**.

In order to utilize the collected data with ML models, they underwent appropriate processing. All data were converted into integer representations, and any instances of missing values were addressed using the previously outlined method. To resolve the issue with the class imbalance in the training set, the oversampling technique SMOTE was used (20). After applying oversampling, each of the three classes in the training data comprised 194 patients. The synthetic samples were created through nearest neighbors (21) with a default value of 5.

The random forest model was chosen as a classifier, and each day for the treatment period of 8 weeks, a model was trained. The model was trained each day using all the data from that day and the preceding days. Each model was validated with a fivefold cross validation. Parallel to the fivefold cross validation, the grid search was used to test different combinations of hyperparameter values to receive optimized values based on the data. The search space comprised four different hyperparameters, namely the number of estimators, maximal depth, splitting criterion and maximal features (17). The search space included variations in the number of estimators, ranging from 50 to 200 in increments of 50. Similarly, the maximal depth explored values of 25, 50, 100, 150, and 200. In terms of maximal features, the options considered were “sqrt” and “log”. and additionally, the splitting criterion encompassed “entropy,” “gini,” and “logloss”. The hyperparameter values that resulted in the best result from the grid search were saved and utilized during the actual training for each of the models.

The scikit learn library was used for the implementation of random forest, cross-validation, linear regression, grid search, and nearest neighbors (22).

Predictions were conducted daily for all patients in the test group. The results yielded three distinct probabilities, each representing the likelihood of a patient’s treatment outcome from the three possible outcomes. To evaluate the model’s ability to predict the treatment outcome, various methodologies were employed. To prevent overfitting in the model, the mean accuracy of the fivefold cross validation with the standard deviation was measured. For the purpose of obtaining a comprehensive assessment of the model’s predictive performance concerning the treatment outcome for the test group, the accuracy was measured for each individual day. In addition to the accuracy metric and to highlight the class imbalances, precision and recall for the classifiers, and also stacked bar plots using certain threshold values for the probability of a patient being successful, partially successful, or unsuccessful, were implemented.

To receive an appropriate threshold for different outcomes, an optimization was carried out. The purpose of the optimization was to determine the threshold that resulted in the largest number of true positives while minimizing the occurrence of false positives. Therefore, quotients between true positives and false positives were calculated for each possible combination of threshold values each day. For all possible combinations, the sum of the quotients from day 1 through day 56 was calculated and the combination with the highest sum (highest true positive-to-false positive ratio) was chosen as the most appropriate threshold combination to receive the best result based on the criterion. Due to our goal of making early predictions regarding treatment outcomes, the thresholds were optimized from the beginning of week 3 to the end of week 5. This was further motivated by the fact that predictions about treatment outcomes in the final weeks held little interest for the patients. In addition, the initial 2 weeks of treatment provided insufficient information for us to make qualified predictions.

### 2.1 Ethical considerations

The study was approved by the Swedish Regional Ethics Authority (2021–00206) and conducted in accordance with the Declaration of Helsinki. The utilization of the data that were provided by patients from the application was cleared in accordance with the General Data Protection Regulation. The identities of the patients were unknown to the researchers.

## 3 Results

### 3.1 Cross validation

**Figure 4:**
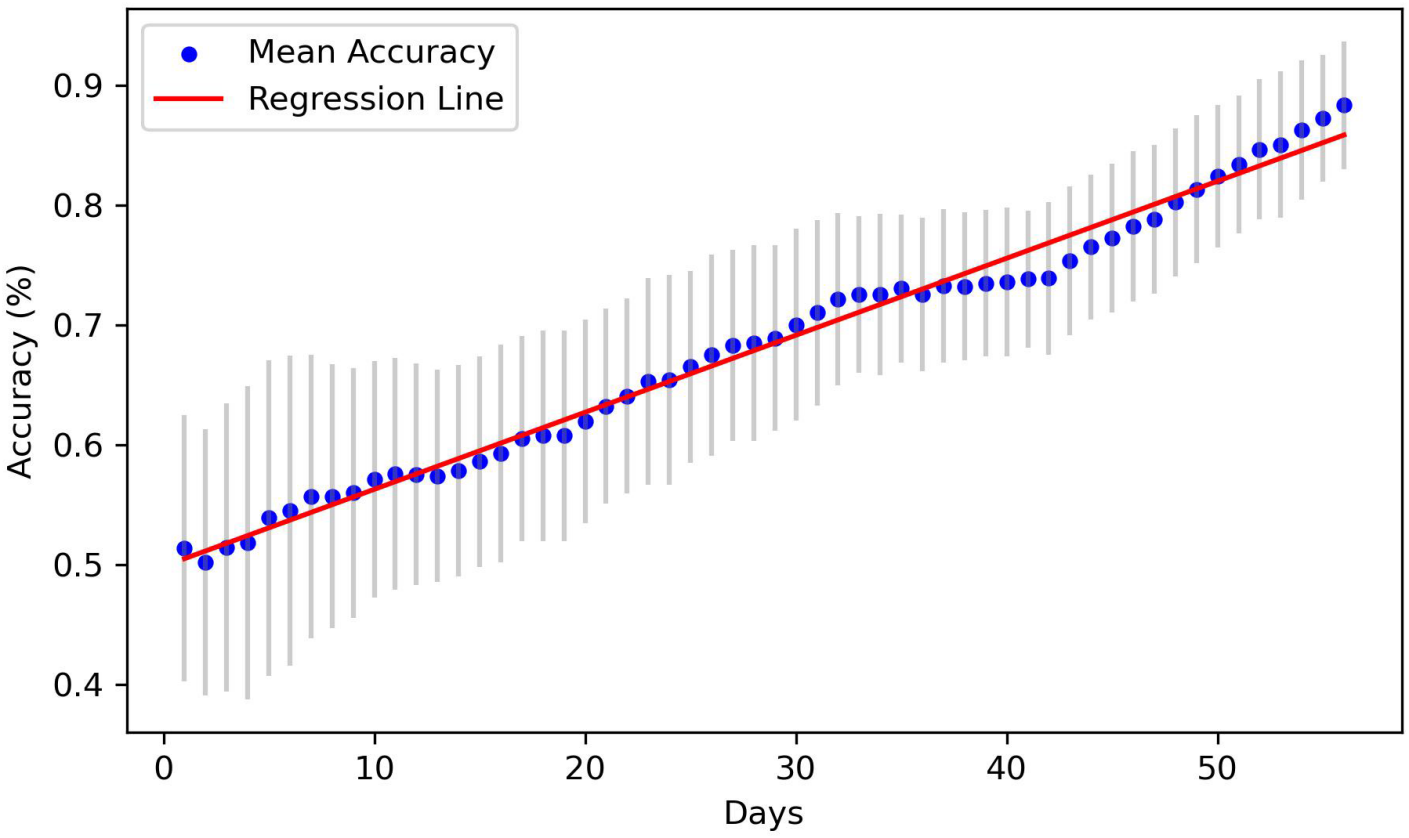
Mean accuracy and standard deviation each day using fivefold cross-validation.

Of the 611 patients who were not considered dropouts, 427 patients were used when training the random forest classifiers. **Figure 4** presents the mean accuracy and standard deviation from the fivefold cross-validation each day. A regression line fitted to the data of the mean accuracy is also presented. The regression line has a slope of 0.0065 and coefficient of determination (*R* ^2^) of 0.98. The interpretation of these metrics is that the mean accuracy is rising in a linear manner, and that the data variability is low. The standard deviation is shown to consistently decrease as more days pass. These results indicate that the model presents reliable results no matter what section of the data frame is used.

### 3.2 Accuracy

**Figure 5:**
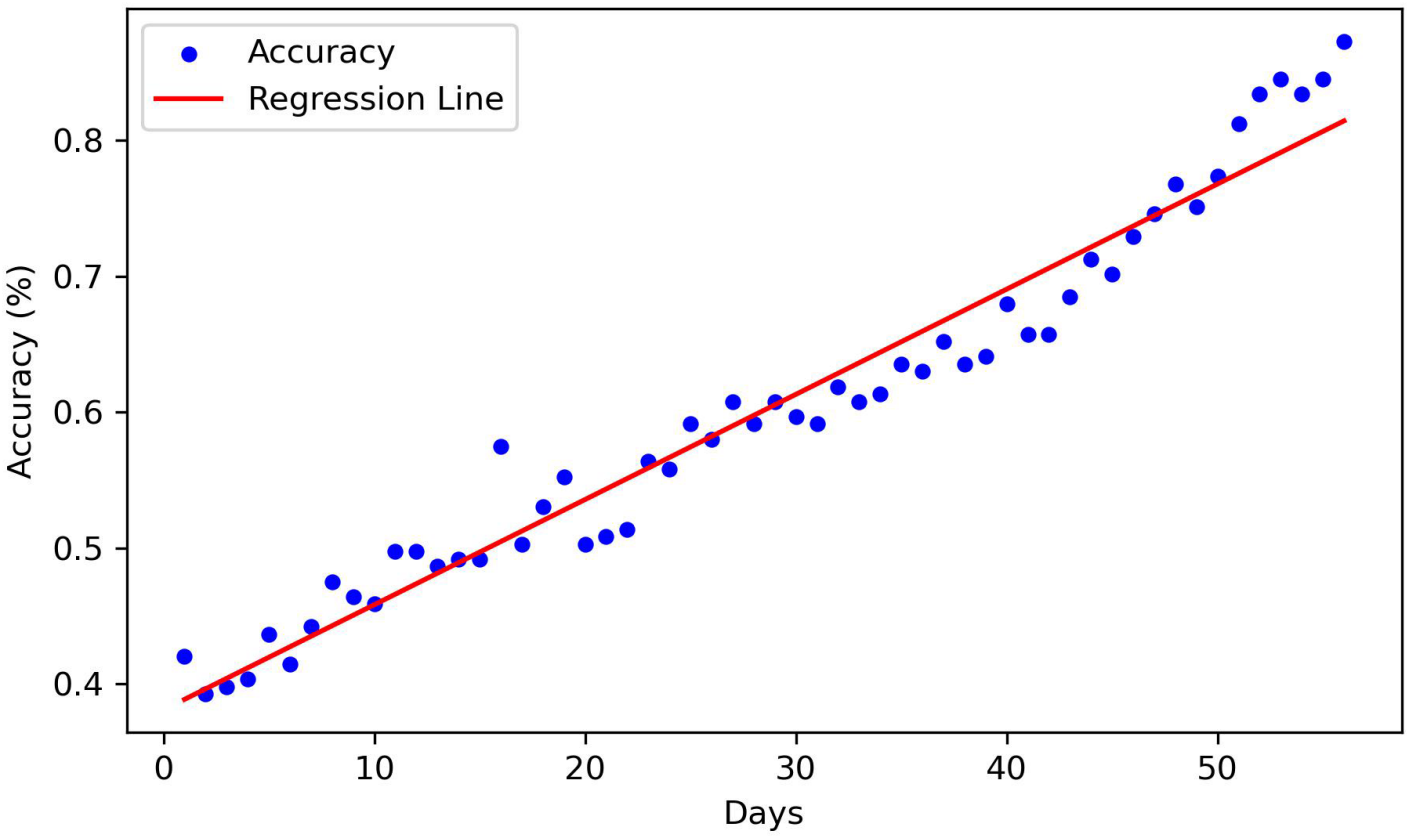
Accuracy of the classifiers each day in predicting the treatment outcome.

Of the 611 patients who completed treatment, the 184 patients who were not included in the training process were used to assess the predictive performance of the trained random forest classifiers. The accuracy values depicted in **Figure 5** were obtained from these predictions. Although day-to-day accuracy fluctuations were noted, an overarching trend revealed a gradual enhancement in the accuracy over time. The average accuracy in week 3 (day 15 to day 21) was equal to 0.51, that in week 4 was equal to 0.59, and that in week 5 was equal to 0.64. The regression line has a slope of 0.0079 and coefficient of determination (*R* ^2^) of 0.95.

### 3.3 Precision and recall

**Figure 6:**
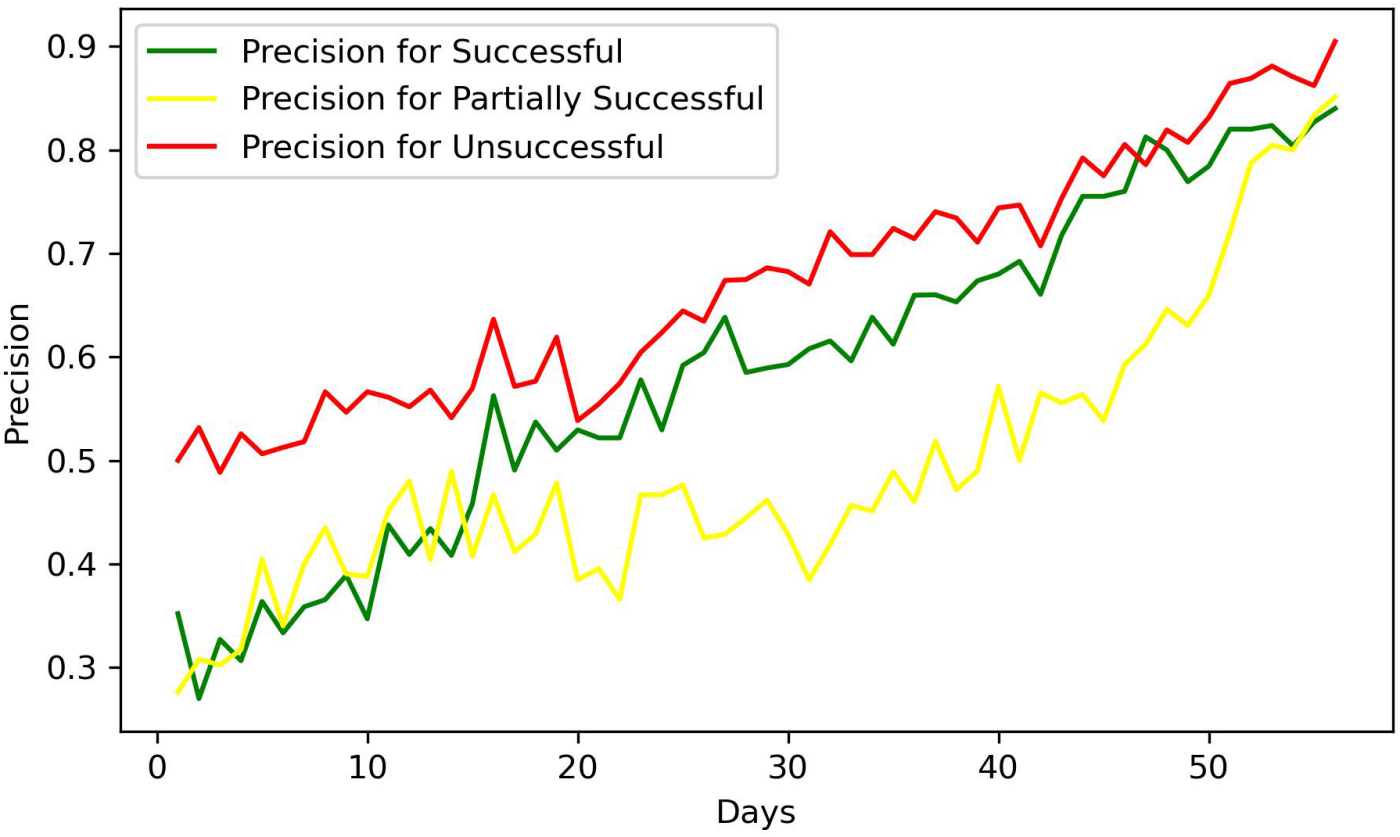
Precision of the classifiers.

**Figure 7:**
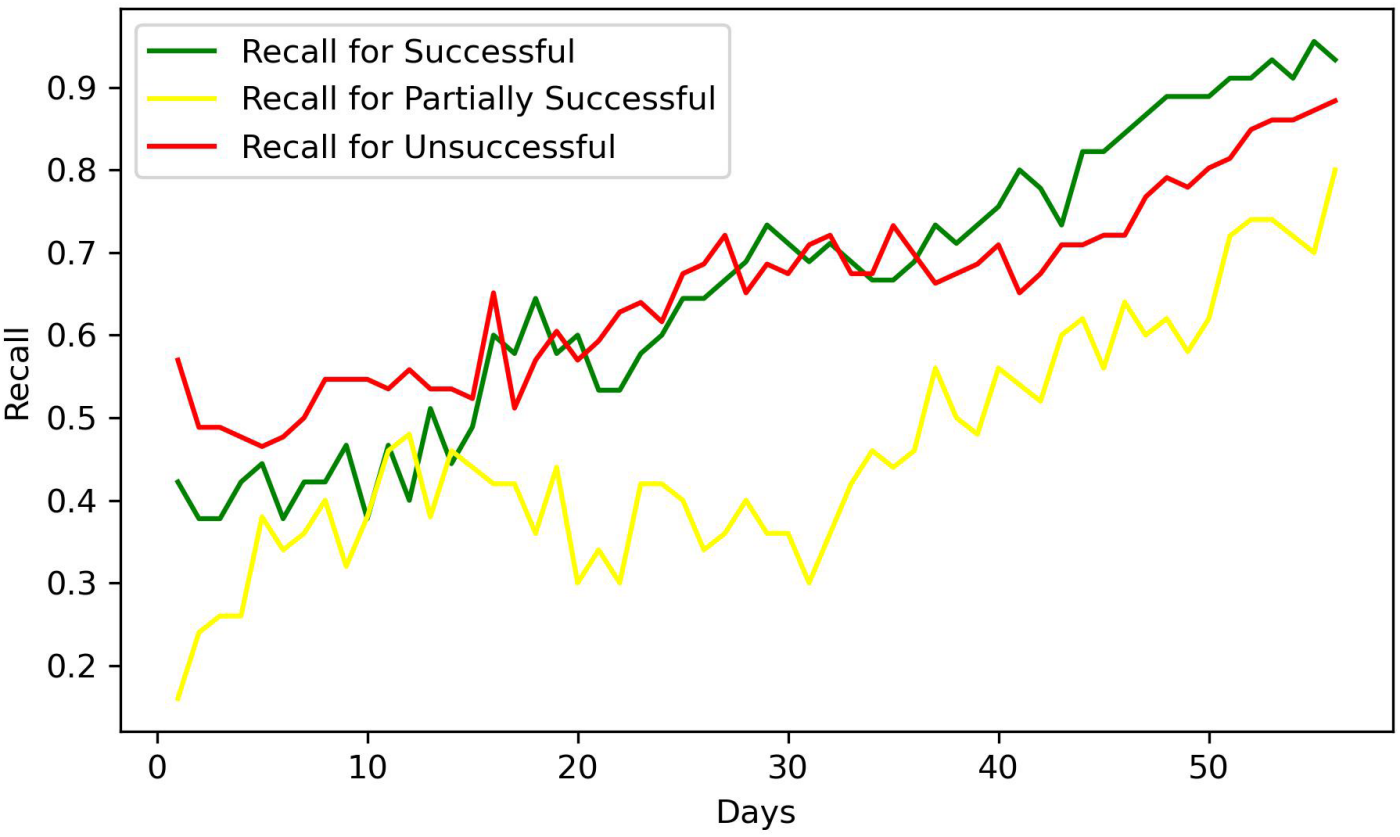
Recall of the classifiers.

**Figures 6**, **7** showcase the precision and recall of the model throughout the 56-day treatment process. Notably, both metrics exhibit consistent improvement across all patient groups during the course of treatment. It is also noteworthy that the precision values for the unsuccessful patient group surpassed those of the other groups throughout the entire treatment duration. In terms of recall, the successful and unsuccessful groups initially exhibited closely aligned values for the majority of the treatment. However, recall for the successful group surpassed that of the unsuccessful group in the final weeks of treatment. Notably, the patient group classified as partly successful demonstrates the lowest scores for both precision and recall.

### 3.4 Patient classification

**Figure 8:**
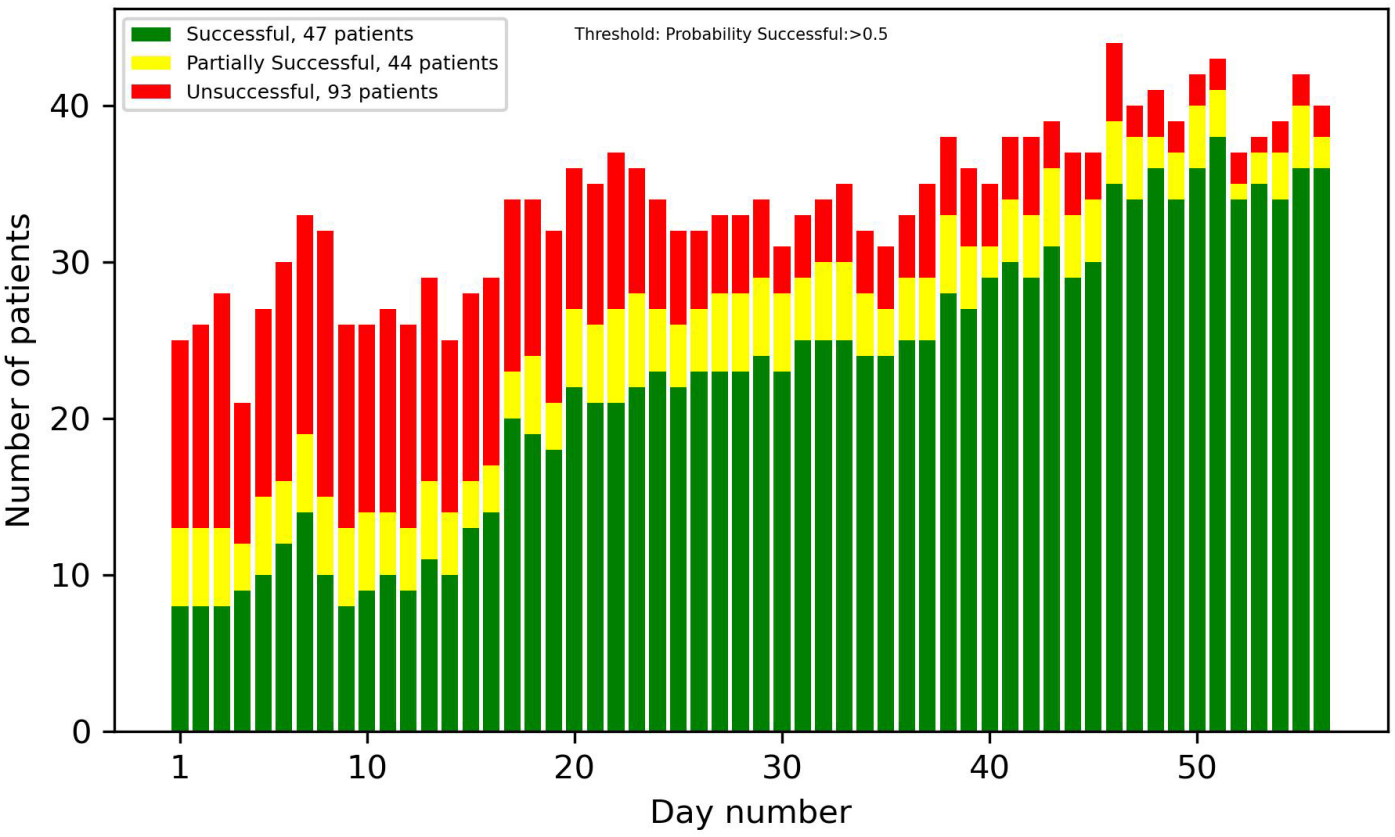
Number of patients predicted to have more than a 50% probability of successful treatment each day.

**Figure 9:**
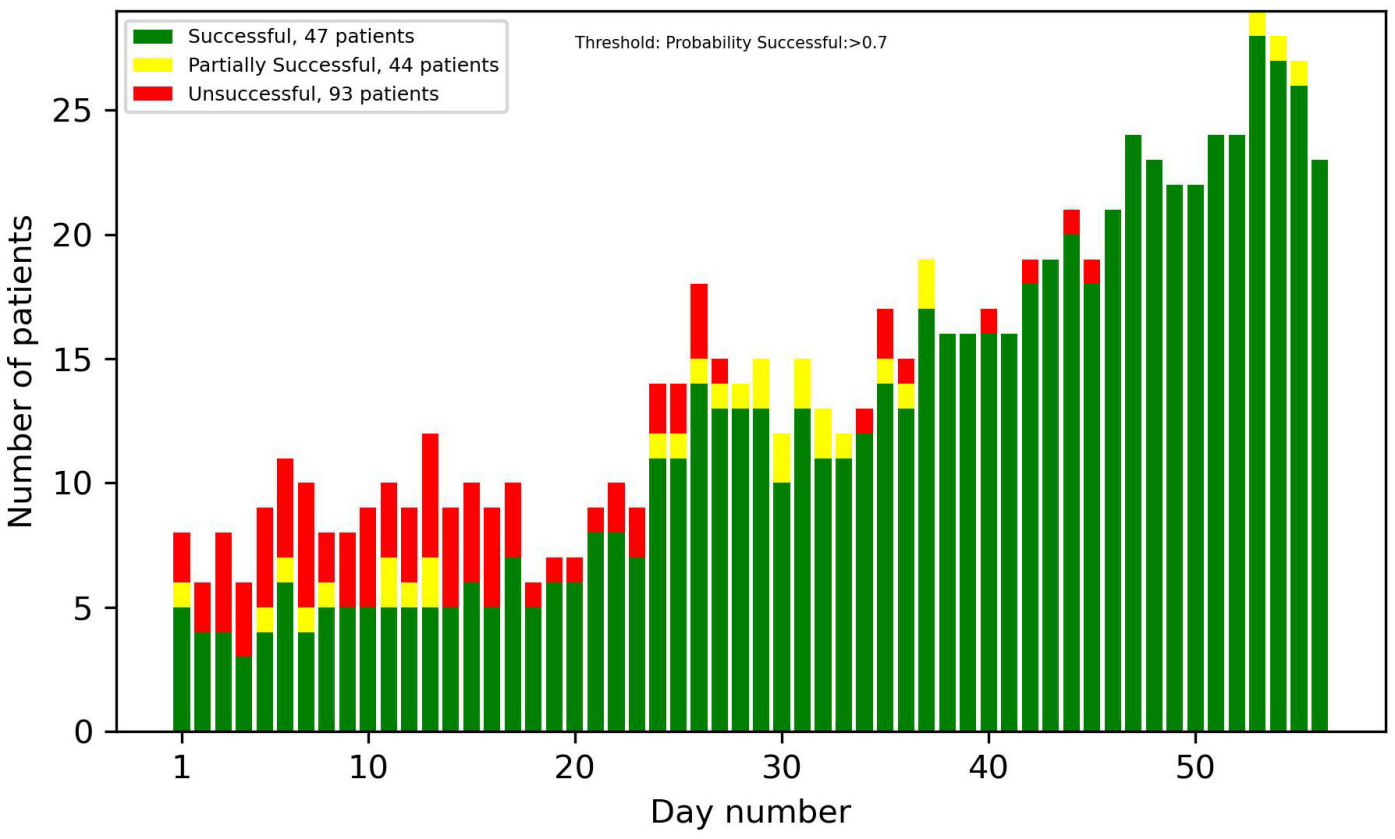
Number of patients predicted to have more than a 70% probability of successful treatment each day.

**Figure 10:**
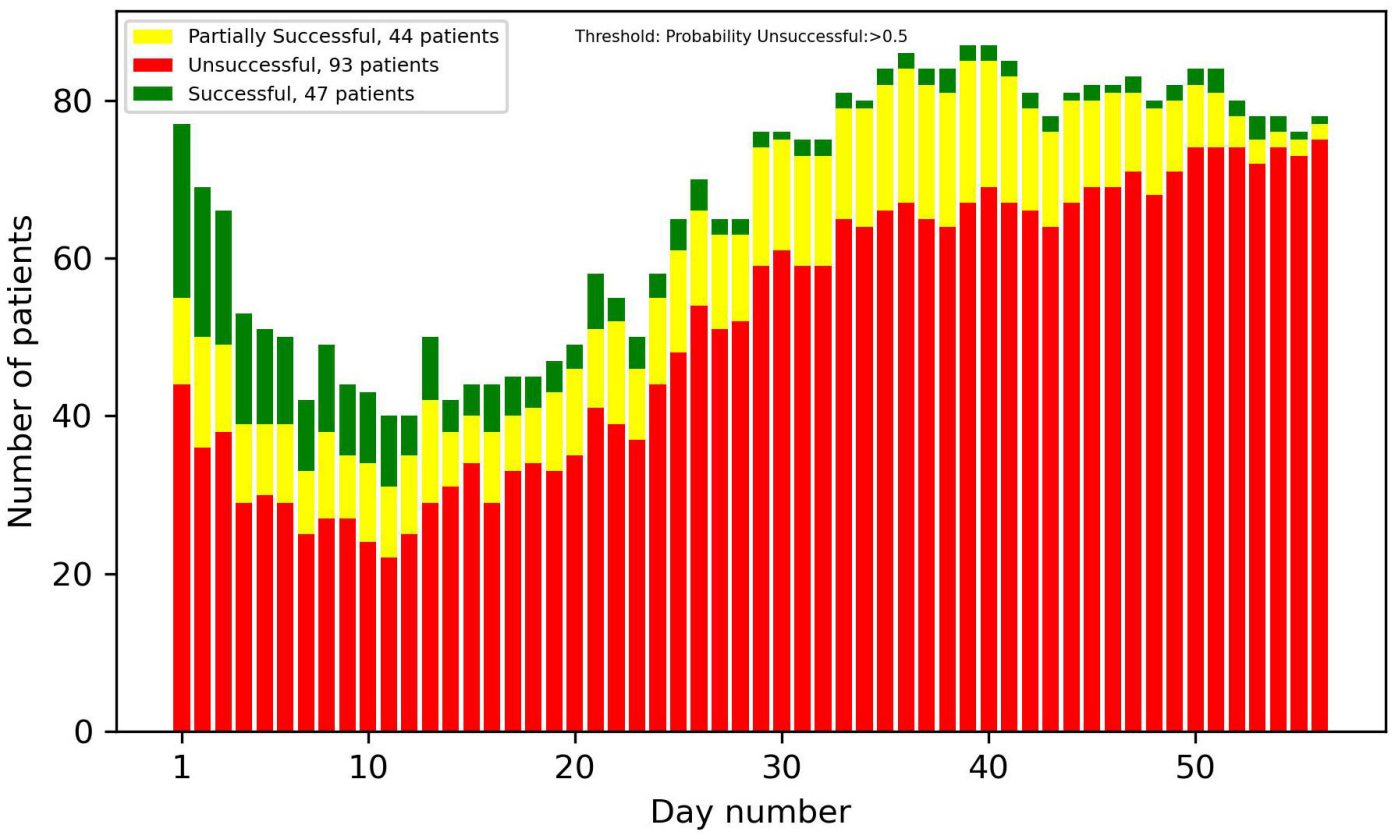
Number of patients predicted to have more than a 50% probability of unsuccessful treatment each day.

**Figure 11:**
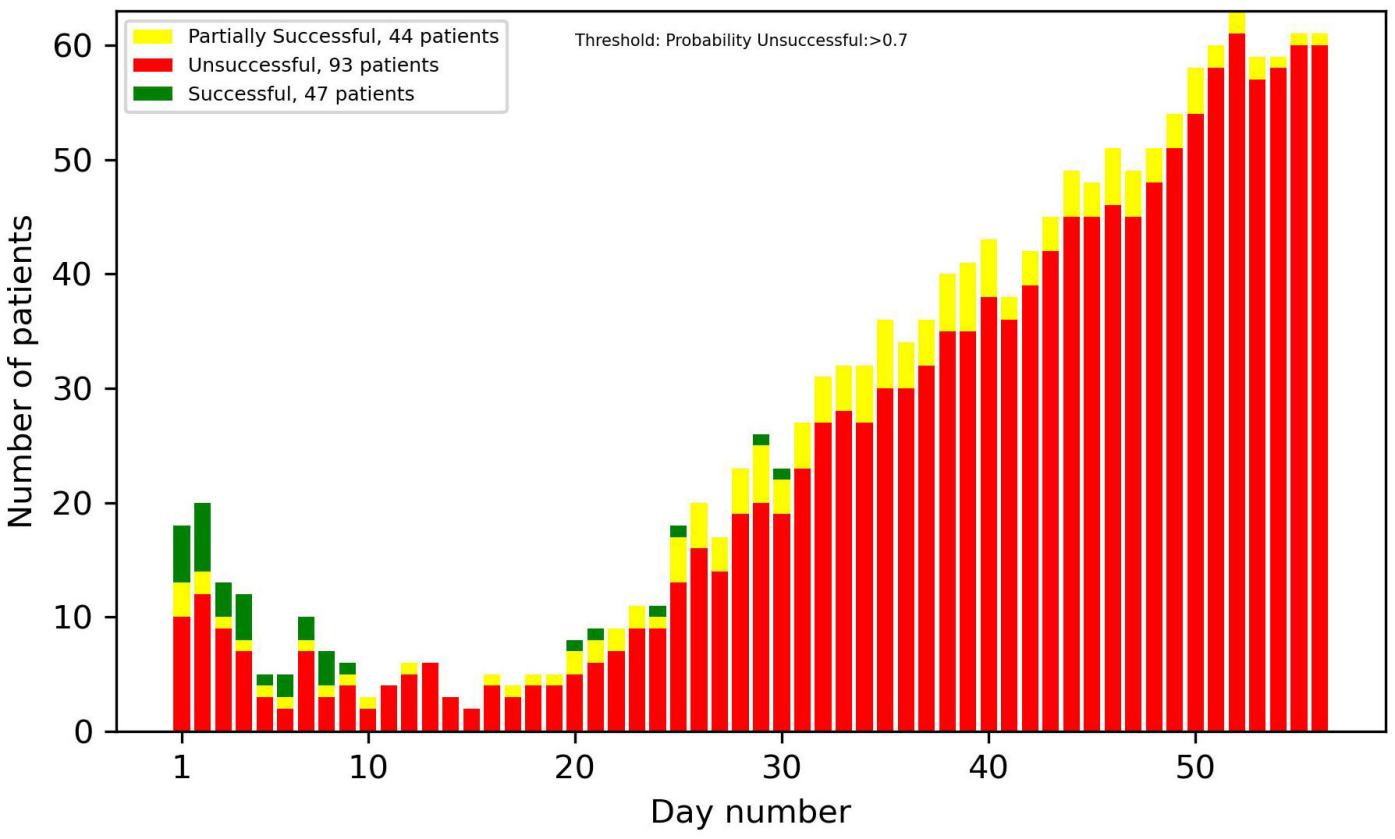
Number of patients predicted to have more than a 70% probability of unsuccessful treatment each day.

**Figure 12:**
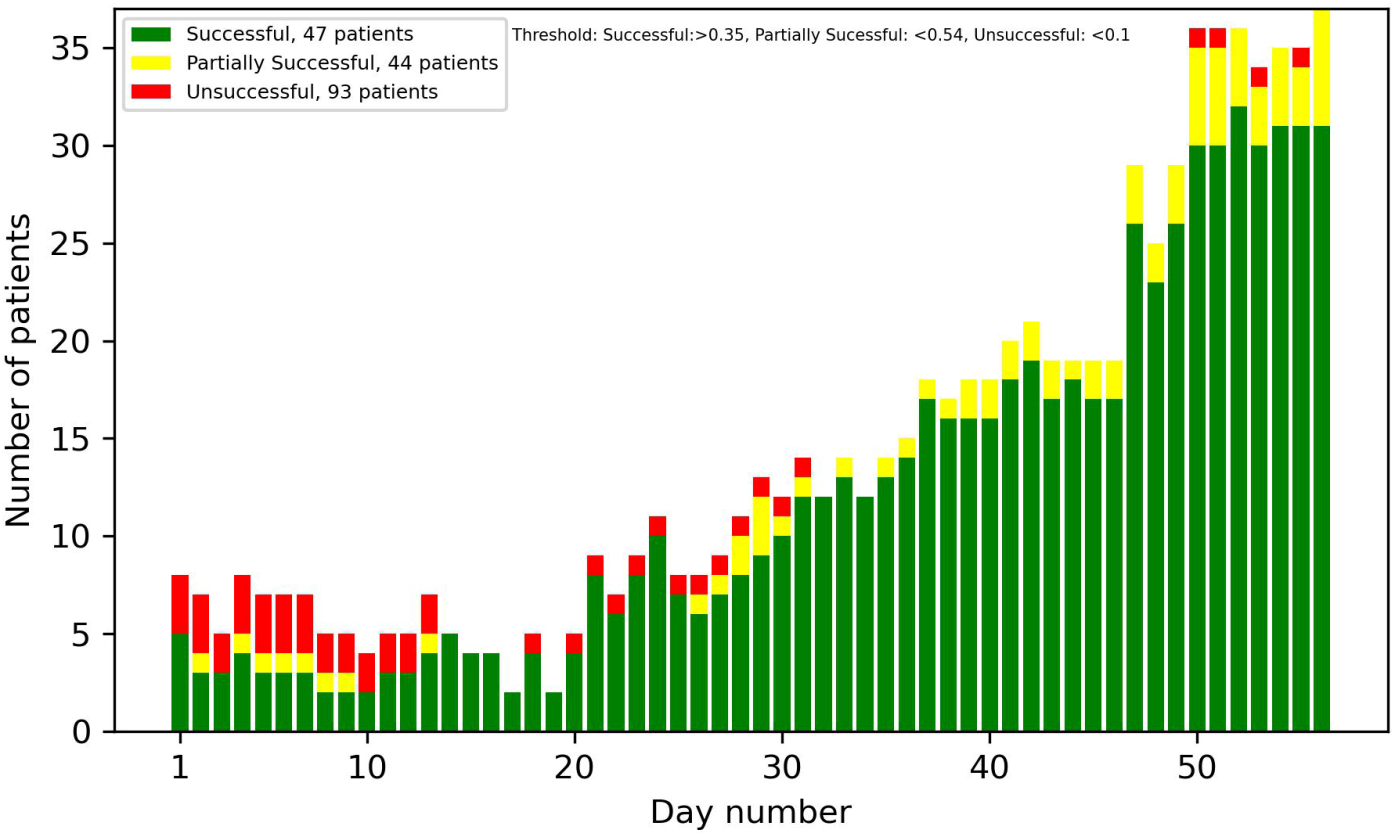
Predictions of successful patients using thresholds optimized for weeks 3–5.

**Figure 13:**
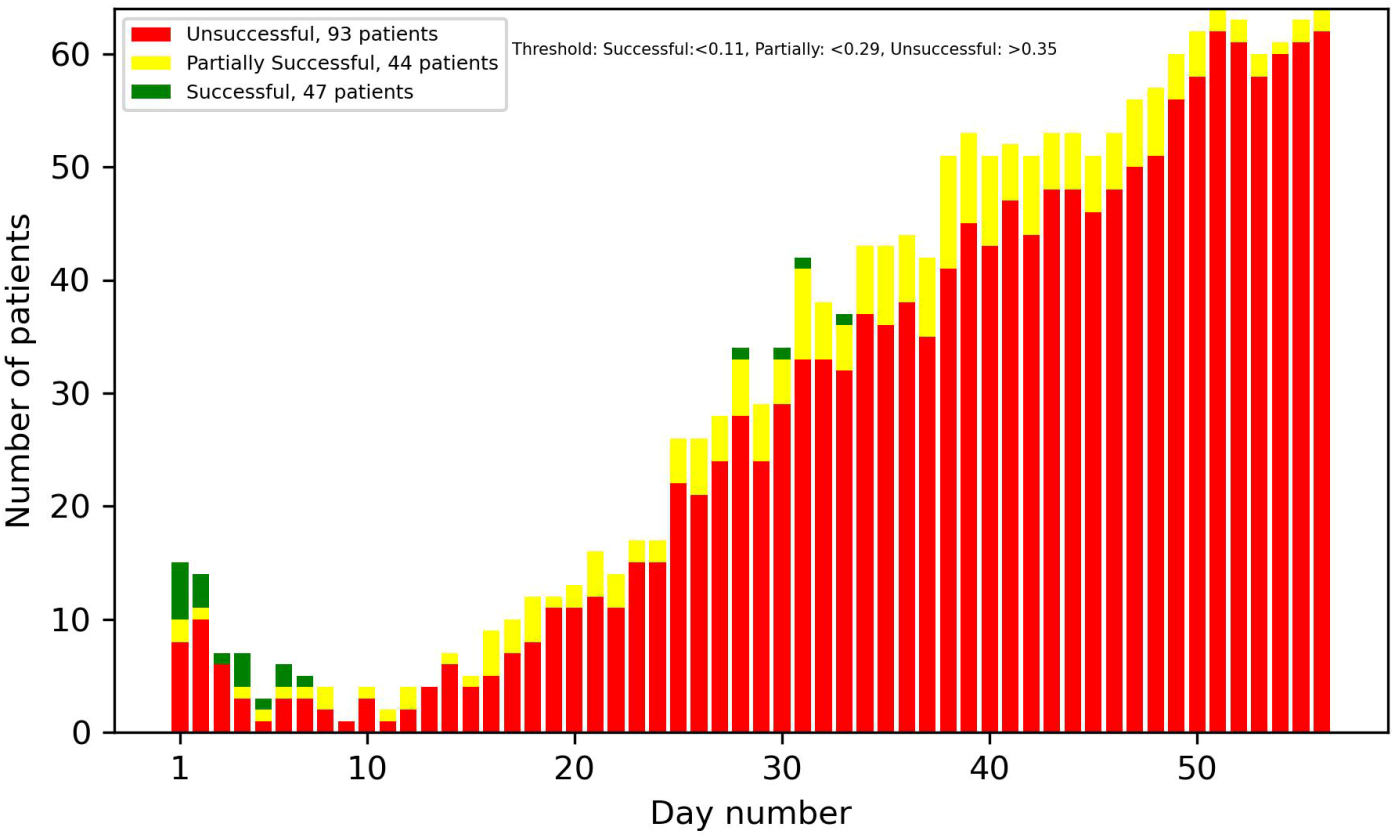
Predictions of unsuccessful patients using thresholds optimized for weeks 3–5.

**Figures 8**-**13** showcase the predictions of patient outcomes using different threshold values for the probability of different outcomes. Out of the 184 patients in the test group, 47 were marked as successful, 44 as partially successful, and 93 as unsuccessful based on the ICCS guidelines. In **Figure 8**, the threshold is set to show patients with a probability larger than 0.5 for a successful treatment, and **Figure 10** shows the same probability, but for an unsuccessful treatment. In these plots, a large number of both true and false positives is observed. As the treatment progresses, the number of false positives decreases, while the number of true positives increases. In **Figures 9**, **11**, the threshold was increased to 0.7, resulting in a large decrease in false positives, and also in a decrease in true positives. It is important to note that the overall amplitude decreases when the threshold values increase.

In **Figures 12**, **13**, optimized thresholds have been applied for predicting unsuccessful and successful patients, spanning from week 3 to the conclusion of week 5. The adjustment of the false positive-to-true positive ratio optimized the outcomes, yielding a nuanced variation compared with the utilization of a fixed threshold at 0.7. The observed outcome manifests as a reduction in the number of false positives coupled with an increase in the number of true positives.

Looking at the first 2 weeks of the plots, there is an uncertainty observed, with a similar ratio of false positives to true positives.

## 4 Discussion

### 4.1 Results

The overall results demonstrate potential in identifying patients at an early stage in the treatment that are on the path of being successful or unsuccessful. From week 2 onwards, there was a noticeable number of patients classified correctly, and this number progressively increased each week. With varying and narrowing the threshold values these can be identified without substantial risk of including false positives. Optimal results in the threshold plots are achieved when classifying unsuccessful patients, as this configuration yielded a larger number of true positives and an enhanced ratio of true positives to false positives. This interpretation of the results was also supported by the precision plot, which measured the ratio of the true positives to all the positives. The considerable factors were the unsuccessful group, which consists of twice as many patients as the successful group. This did not only mean that the amplitude was higher, but that the probability of an unsuccessful patient acting as a false positive in the successful predictions was also higher, and therefore the successful plots had a smaller amplitude and a larger number of false positives. In most cases though, as seen in the results, the false positives were partially successful patients. This is not as big of a problem as, for instance, a successful patient being predicted to be unsuccessful.

Taking into account every prediction plot and recall analysis, the initial 2 weeks exhibit limited predictive efficacy. Consequently, the interpretations of predictions during this period should be approached with caution. If these predictions were to be incorporated into a patient portal for tracking treatment progress, it might not be ideal to display the predictions before day 14, due to their reduced reliability.

### 4.2 Clinical utility

It is worth noting that a significant drawback of enuresis alarm therapy is the substantial effort and sleep disturbance it imposes on both the patient and their family (2). One session of several months of unsuccessful treatment could make the family disinclined to ever try the therapy again. Furthermore, adherence to therapy is another problem with the enuresis alarm. Perhaps not even half of the users will manage to continue therapy for every night, as instructed, for more than 1 or 2 weeks (9). Presumably, many of the non-adherent patients would have become dry if they continued. The clinical usefulness of these predictions is thus clear. The likelihood of the individual patient achieving dryness can be communicated either to the healthcare provider or to the patient and their family. This way, patients with a significant chance of treatment success can be encouraged to adhere to therapy, whereas those with a low chance of becoming dry can be advised to stop treatment and seek other help, thus not having to undergo several more weeks or months of futile efforts.

A difficult question that arises when predictions with the model are made is what level of inaccuracy is tolerable. For instance, when predicting which patients will have an unsuccessful treatment, only a small share of patients can be correctly predicted during the initial weeks of treatment, if no to very few false predictions are accepted. Although this approach may initially appear optimal, it raises ethical concerns about subjecting children with only a minimal likelihood of a successful treatment to 8 or more weeks of the alarm treatment as opposed to exploring alternative treatment options. The correct approach might be a compromise, where more true predictions are made early, at the cost of also having some false predictions. The optimal approach regarding these questions is something that should be discussed among healthcare professionals and enuresis experts.

### 4.3 Real-world validity and data trustworthiness

When assessing the model’s efficacy and potential applications, real-world relevance was crucial. This involves how accurately the model’s predictions aligned with the actual outcomes for patients undergoing treatment. The training and evaluation of the model relied on treatment data recorded in the Pjama application’s calendar. However, the presence of missing days in some patients’ calendars posed some challenges. The primary concern was the potential bias when users left days unfilled, influenced by factors such as wet nights receiving more attention than dry nights. Illustrating this, a hypothetical patient consistently reporting wet nights but no dry nights could achieve success in reality but not be classified as such. Another aspect of this issue involves our decision to categorize patients with 1 or 2 missing days within a 2-week span as successful if all other days were dry. Allowing 0 or 1 missing days was unrealistic due to the high alarm treatment response rate. Conversely, permitting more missing days would create different issues. To strike a balance, allowing 2 missing days was deemed optimal and minimizes incorrect patient categorizations in terms of both success and failure.

### 4.4 Potential improvements of the model

While the results are promising, there are still improvements that could be made. One potential improvement could result from an increase in the amount of available data. As time passes and more patients use the application, two aspects of the model could improve. First, more patients could be used for training the algorithm. Second, the choice of which patients are used for training and testing the model could be more selective if more patients were available. By excluding patients with many missing days, the real-world validity of the marking of patient outcomes and the accuracy of predictions could also potentially increase. The dataset may also expand through the collection of more data from each patient. New questions could be introduced in the registration, or users may be presented with additional questions regarding information about each night. Some questions might be strong predictors of a certain outcome, while others could have no effect on the predictive performance of the model. Questions should thereby be added with care. In the worst case, new questions could have the opposite effect, since more questions each day might discourage families from filling in the calendar, leading to more missing days. If more questions are added, they should not have a negative impact on the user experience, and be related to the factors known or suspected to affect the treatment outcome.

## 5 Conclusion

The prediction of the treatment outcome has great potential in making the treatment of enuresis more efficient. When the model is able to identify certain patients early and without risks of involving false positives, correct treatment interventions, guidelines, and motivational pushes can be provided to optimize the treatment and speed up the results. In the near future, when more patients have used the Pjama application, the results will likely improve even more. This could revolutionize the treatment of enuresis as a whole, and also open the door for other improvements in the field.

## Data availability statement

The datasets presented in this article are not readily available because the data belongs to the company Pjama AB. Requests to access the datasets should be directed to torbjorn.gardenfors@pjama.se.

## Ethics statement

The studies involving humans were approved by Swedish Regional Ethics Authority. The studies were conducted in accordance with the local legislation and institutional requirements. Written informed consent for participation was not required from the participants or the participants’ legal guardians/next of kin in accordance with the national legislation and institutional requirements.

## Author contributions

K-AJ: Conceptualization, Data curation, Formal Analysis, Methodology, Software, Visualization, Writing – original draft, Writing – review & editing. EA: Conceptualization, Data curation, Formal Analysis, Methodology, Software, Visualization, Writing – original draft, Writing – review & editing. TN: Conceptualization, Formal Analysis, Supervision, Validation, Writing – review & editing. TG: Conceptualization, Data curation, Investigation, Methodology, Project administration, Resources, Supervision, Writing – review & editing. CB: Conceptualization, Formal Analysis, Funding acquisition, Supervision, Validation, Writing – review & editing.
